# Time-course investigation of *Phytophthora infestans* infection of potato leaf from three cultivars by quantitative proteomics

**DOI:** 10.1016/j.dib.2015.11.069

**Published:** 2015-12-17

**Authors:** Mia Kruse Guldstrand Larsen, Malene Møller Jørgensen, Tue Bjerg Bennike, Allan Stensballe

**Affiliations:** a Department of Chemistry and Bioscience, Aalborg University, Aalborg, Denmark; b Department of Clinical Immunology, Aalborg University Hospital, Urbansgade 32-36, DK-9000 Aalborg, Denmark; c Department of Health Science and Technology, Aalborg University, Aalborg, Denmark

## Abstract

Potato late blight is one the most important crop diseases worldwide. Even though potato has been studied for many years, the potato disease late blight still has a vast negative effect on the potato production [1], [2], [3]. Late blight is caused by the pathogen *Phytophthora infestans* (*P. infestans*), which initiates infection through leaves. However, the biological activities during different stages of infection are poorly described, and could enable novel or improved ways of defeating late blight infection [4]. Therefore, we investigated the interactions between *P. infestans* (mixed strain culture) and potato *(Solanum tuberosum*). Three commercially available field potato cultivars of different resistance to late blight infection; Kuras (moderate), Sarpo Mira (highly resistant) and Bintje (very susceptable) were grown under controlled green house conditions and inoculated with a diversity of *P. infestans* populations.

We used label-free quantitative proteomics to investigate the infection with *P. infestans* in a time-course study over 258 h. Several key issues limits proteome analysis of potato leaf tissue [5], [6], [7]. Firstly, the immense complexity of the plant proteome, which is further complicated by the presence of highly abundant proteins, such as ribulose bisphosphate carboxylase/oxygenase (RuBisCO). Secondly, plant leaf and potato, in particular, contain abundant levels amounts of phenols and polyphenols, which hinder or completely prevent a successful protein extraction. Hitherto, protein profiling of potato leaf tissues have been limited to few proteome studies and only 1484 proteins have been extracted and comprehensively described [5], [8], [9]. We here present the detailed methods and raw data by optimized gel-enhanced label free quantitative approach. The methodology enabled us to detect and quantify between 3248 and 3529 unique proteins from each cultivar, and up to 758 *P. infestans* derived proteins. The complete dataset is available via ProteomeXchange, with the identifier PXD002767.

## Graphical abstract

**Figure:**
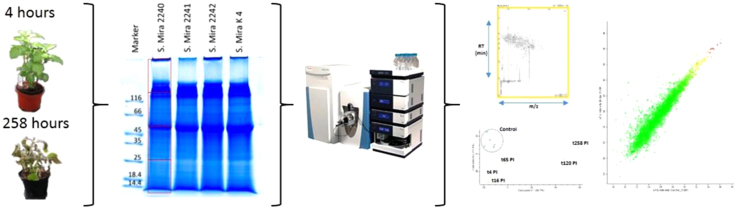

**Specifications Table**

**Table:** 

Subject area	*Biology*
More specific subject area	*Plant proteomics*
Type of data	*Raw files and text/excel files*
How data was acquired	*Mass Spectrometry Liquid Chromatography*
*High-resolution/high-accuracy mass spectrometer*
*system was used: Q Exactive (Thermo Scientific)*
Data format	*Raw and analyzed data.*
Experimental factors	*Proteome analysis of three potato cultivars following time-course infection with late blight (Phytophthora infestans)*
Experimental features	*Three potato cultivars were infected with Phytophthora infestans in a time-course over 258 h. Leaf proteins were extracted and analyzed by GelMS using 298 runs of 2.5 h UPLC-OrbitrapMS prior to label-free proteome analysis using MaxQuant.*
Data source location	*Aalborg, Denmark*
Data accessibility	*The MS proteomics data have been deposited to the ProteomeXchange Consortium via the PRIDE partner repository with the dataset identifier PXD002767. Direct download link:*http://www.ebi.ac.uk/pride/archive/projects/PXD002767

## Value of the data

• Highly optimized protein extraction method for potato leaf significantly reducing effects of polyphenols and Rubisco.
• Most extensive proteomic analysis of potato leaf so far (>4000 unique proteins).
• Time-course infection of three cultivars of wide resistance towards late blight enabling study of biological response to infection.

## 1 Experimental design, materials and methods

### 1.1 Collection of potato leaf samples

Potato plants of the three cultivars Kuras, Bintje, and Sarpo Mira were grown over the summer of 2008 in a big greenhouse at Landbrugets Kartoffelfond (LKF) Vandel, Denmark. The potato plants were divided into five groups of *Phytophthora infestans* inoculated plants and four groups of control plants. Each group contained three plants per cultivar. The inoculation was obtained by bulk spraying a poly-strain inoculum (sporangial suspension) derived from late blight strains collected in Denmark in 2007 on the plants. In order to ensure infection, a high humidity was maintained by covering the plants with plastic overnight. The same procedure was executed for the control plants, which were however sprayed with water instead of *P. infestans* inoculum. For each cultivar, five different sampling time points were selected: 4, 16, 65, 120, and 258 h after spraying. One control and one inoculated group were sampled per time point by taking three replicates per group. One control group was used for two samplings. For each replicate five to six leaves with varying size were sampled and immediately snap-frozen in liquid nitrogen and later stored at −80 °C at Aalborg University, Denmark.

### 1.2 Extraction of from potato leaf

All solutions and samples were kept cold under the entire protein extraction procedure either by using ice or liquid nitrogen. Three to five leaves from one time point were added to a teflon shaking flask (Sartorius, Goettingen, DE) containing a grinding ball (B. Braun, Mannheim, DE). The container was afterwards strapped on to a micro-dismembrator (B. Braun, Mannheim, DE) and the leaf material was homogenized by shaking at 2000 rounds per minute (rpm) for 1 min. 200 mg leaf powder was weighted into a 1.5 mL centrifuge tube (Beckman, Palo Alto, US) containing 500 µL pre-cooled extraction buffer (50 mM HEPES-KOH (pH 7.5), 10 mM potassium acetate, 5 mM magnesium acetate, 1 mM EDTA, 0.5 M tris(2-carboxyethyl)phosphine (TCEP), plant protease inhibitor cocktail (Sigma-Aldrich, Steinheim, DE) containing AEBSF, 1,10-Phenanthroline, Pepstatin A, Leupeptin, Bestatin, and E-64 and lastly a phosphatase inhibitor cocktail (Roche PhosStop). In order to be completely mixed, the leaf sample solution was vortex and then grinded with a pestle. Afterwards, an extra 500 µL extraction buffer was added. The sample was centrifuged in a Sorvall Discovery 90 ultra-centrifuge (Kendro Laboratory Products) at 18.000 rpm (34.000*g*) at 4 °C for 10 min. The yellow/light green supernatant (soluble proteins) was transferred to a new tube and used further on, while the dark green pellet (insoluble proteins) was stored in −80 °C.

### 1.3 Sample preparation and fractionation

The protein concentration of the leaf supernatants was determined by a Bradford assay using ovalbumin (MP biomedicals, FR) as standard. All the samples and standards were assayed in triplicate and diluted using 50 mM phosphatebuffer. Ten µL supernatant, diluted supernatant or standard was used for each quantification and mixed with 250 µL Bradford reagent (0.05% (w/v) Brilliant Blue G-250, 25% (v/v) ethanol (Kemtyl, Køge, DK), and 50% (v/v) phosphoric acid (Merck Millipore, Darmstadt, DE). After 5 min, the resulting color change of the samples was measured at 600 nm using an Infinite®M1000 PRO Tecan (Tecan, Salzburg, AT).

SDS-PAGE was used to separated and fractionate proteins found in the different leaf samples. Supernatant (24 µL) from the inoculated samples or a mixture of the supernatant from the three replicates (8 µL of each) of the corresponding control was mixed with 6 µL 5x reducing SDS sample buffer (0.25 M Tris–HCl (pH 6.8), 5% (w/v) SDS, 50% (v/v) glycerol (Saveen Werner AB), 0.25 M DTT and a couple of bromphenol blue corns) and boiled for 10 min at 100 °C. After boiling, the samples were centrifuged at 14.000 g for 2 min and used for gel electrophoresis with 12% Mini-Protean TGX gels (Biorad, US). The gel electrophoresis was performed at 70 V for 10 min and afterwards at 200 V for 15–20 min (until the sample line had reached ~2/3 of the gel). The gels were stained with Coomassie Brilliant Blue (0.2% solution in 50% ethanol, 7% acetic acid) and destained in 8% ethanol, 5% acetic acid.

In-gel digestion was performed as previously described with few modifications [9]. Each sample lane in the SDS-gels were parted into four big gel slices (top 1–4 bottom), giving 80 gel samples for each potato cultivar (5 time points*4 samples (three inoculated and one control)*4 gel pieces). Each of these four gel samples for one supernatant samples were further chopped into small cubes with a razor blade and transferred to 0.5 mL Protein LoBind tubes (Eppendorf, Hamburg, DE). The gel pieces were washed by incubation for 5 min in 100 µL 0.1 M NH_4_HCO_3_ and for 15 min in 0.1 M NH_4_HCO_3_/acetonitrile (1:1), afterwards both washing steps were repeated. The gel pieces were covered by an appropriate amount of acetonitrile (Panreac, Barcelona, ES) in order to dehydrate the pieces. After the gel pieces had shrunk, the acetonitrile was removed and if residual staining was still observed the washing cycle was performed once more. The samples were reswelled in 125 µL 10 mM DTT in 0.1 M NH_4_HCO_3_ and incubated at 56 °C for 45 min. The samples were cooled to room temperature and the liquid was replaced with 100 µL 55 mM iodacetic acid in 0.1 M NH_4_HCO_3_, incubated in the dark for 30 min, and a washing procedure was carried out as before. After dehydration, the gel pieces were reswelled in 120 µL digestion buffer (50 mM NH4HCO3, pH 8 and 12.5 ng/µL of sequencing-grade modified porcine trypsin (Promega, Madison, US)). The samples were incubated on ice for 45 min and the supernatant was removed and replaced with 120 µL 50 mM NH_4_HCO_3_, pH 8, and incubated overnight at 37 °C. The following day the peptides were extracted from the gel pieces. The overnight supernatant was transferred to a new 0.5 mL Protein LoBind tube and saved. One hundred µL 5% formic acid was added to the gel pieces and after 15 min 100 µL acetonitrile was added and the samples were incubated an additionally 15 min. The supernatant was pooled with the overnight supernatant and the procedure was repeated. The pooled samples were dried in a Centrivap Concentrator until no solution was left. The samples were then re-dissolved in 20 µL 0.1% TFA with either 10 fmol/µL MassPREP Enolase digestion standard (Waters, SwissProt P00924) or 10 fmol/µL MassPREP Bovine Serum Albumin (BSA) digestion standard (Waters, SwissProt P02769). The enolase digestion standard was used for all Sarpo Mira samples, while the BSA digestion standard was used for both Bintje and Kuras samples.

### 1.4 LC−MS/MS analysis

UPLC was performed on a Dionex Thermo Scientific UPLC designed to operate at ultra-high pressures up to 1000 bars with a commercial pre-column set up also from Dionex Thermo Scientific. Peptides from an in gel digestion sample (5 µL (1/4 of the sample)~12.5 µg) were first loaded on to a 2 cm long pre-column with 100 µm inner diameter packed with 5 µm C18 particles and further analyzed by a 50 cm separation-column with 75 µm inner diameter packed with 2 µm C18 particles. The columns were operated under a constant temperature of 40 °C. The reversed phase chromatography was executed with a binary buffer system consisting of buffer A (0.1% (v/v) formic acid and 0.005% (v/v) heptafluorobutyric acid in ultrapure water) and buffer B (90% (v/v) acetonitrile, 10% (v/v) Ultrapure water, 0.1% (v/v) formic acid and 0.005% (v/v) heptafluorobutyric acid). The peptide mixture was loaded onto the pre-column with 2% buffer B at a flow rate of 4 µL/min. After loading, the Sarpo mira peptide samples were separated and eluted by first a gradient of 4–15% buffer B over 6 min and afterwards a long gradient of 15–55% buffer B over 114 min. The peptide samples of both Bintje and Kuras were separated using an optimized LC method with first a gradient of 4–20% buffer B over 3 min and then a 114 min gradient of 20–45% buffer B. In both methods, the eluted peptides entered the Q Exactive with a flow rate of 0.3 µL/min. The LC was coupled to a Q Exactive mass spectrometer (Thermo Scientific) via an electrospray source (Proxeon, Thermo Scientific).

The MS data was obtained using a data-dependent method with full scans (MS) obtained from range 325–2000 m/z at a resolution of 70.000 at m/z 200 (transient time at 120 ms for the Sarpo mira samples and at 250 ms for Bintje and Kuras samples). Up to the top 12 most abundant precursor ions from each survey scan were in the Sarpo mira samples selected with an isolation window of 1.2 m/z and fragmented with higher energy collisional dissociation (HCD) fragmentation with normalized collision energies at 25. For both Bintje and Kuras samples, the precursor ions were selected with an isolation window of 2 m/z and fragmented with normalized collision energies at 30. The MS/MS scans were furthermore acquired with a resolution of 17.500 at m/z 200 (transient time at 60 ms for the Sarpo mira samples and at 80 ms for Bintje and Kuras samples). The automatic gain control (AGC) used for analyzing Sarpo mira samples in both full- and MS/MS scans was 1e^6^, while AGC for analyzing Bintje and Kuras samples was 3e^6^ for full scans and 2e^5^ for MS/MS scans. Repeated sequencing of peptides was kept at a minimum by dynamic exclusion of the sequenced peptides for 30 s (Sarpo mira samples) or 40 s (Bintje and Kuras samples). Furthermore, the fragmentation event was for the Bintje and Kuras samples triggered at highest intensity of the MS peak by using an Apex trigger from 1 to 20 s.

### 1.5 Proteomic data analysis

The raw data-files from potato leaf analyzed on the Q Exactive were searched using MaxQuant 1.5.3.8 (separate cultivars) and 1.5.3.28 (all cultivars) against two databases containing UniProtKB Potato reference proteome AUP000011115 (53104 sequences, july 2015) and UniProtKB *P. infestans* T30-4 (17609 sequences, july 2015). All standard settings were employed with carbamidomethyl(C) as a static modification and deamidation (NQR), oxidation (M), and protein N-terminal acetylation were included as variable modifications. Label-free quantitation of all proteins was performed in MaxQuant based on integrated precursor intensities using default settings, experiments and fractions settings to combine the GelMS raw data [11]. Time-course control samples and time-course *P. infestans* infected samples were searched as independent replicates linked by option "experiment" and the Gel-MS fractions were linked using the option "fraction". This enables statistical analysis based on the search output files as provided in the ProteomXchange and described in detail in Table 2.

## 2 Data

The proteomics raw data and MaxQuant result-files from the analysis have been deposited to the ProteomeXchange Consortium via the PRIDE partner repository with the dataset identifier PXD0002767 [11], [12], [13], [14], and can be downloaded directly (http://www.ebi.ac.uk/pride/archive/projects/PXD002767). Table 1 contains the list of submitted proteomics raw-datafiles and information regarding sample types, species, digestion protocol, and MS system. Table 2 contains the list of submitted analysis result files and a short description of the content. In our protein extraction yield data, the GelMS protocol was the most efficient, yielding the highest number of identifiable proteins in potato leaf. Therefore, we conducted the comprehensive analysis of late blight infected potato leaf from three different cultivars (Bintje, Sarpo Mira and Kuras), analyzed in biological triplicates on a Q Exactive MS (Thermo Scientific).

## Funding sources

The Ministry of Food, Agriculture and Fisheries of Denmark is acknowledged for Grant no. 2101-07-0116. The Obelske family foundation, the Svend Andersen Foundation and the SparNord foundation are acknowledged for grants to the analytical platform enabling this study.

## Figures and Tables

**Table 2:** Description of search file-names in the ProteomeXchange repository PXD002767 based on MaxQuant [11]. Each zip contains a range of files where the four most relevant are described above.

**Search Files Filename**		**Description of content**
MQ_Search_Kuras.zip		MaxQuant result folder “txt” of Q Exactive GelMS potato leaf samples, search against the Uniprot reference proteome databases for potato (AUP000011115) and late blight (AUP000006643).
MQ_Search_Bintje.zip		MaxQuant result folder “txt” of Q Exactive GelMS potato leaf samples, search against the Uniprot reference proteome databases for potato (AUP000011115) and late blight (AUP000006643).
MQ_Search_Sarpomira.zip		MaxQuant result folder “txt” of Q Exactive GelMS potato leaf samples, search against the Uniprot reference proteome databases for potato (AUP000011115) and late blight (AUP000006643).
MQ_Search_all_cultivars.zip		MaxQuant result folder “txt” of Q Exactive GelMS potato leaf samples from all cultivars, search against the Uniprot reference proteome databases for potato (AUP000011115) and late blight (AUP000006643).
**The MaxQuant output in folder "txt" contains a range of files containing important search information. Below files are the most commonly used for post-identification analysis and quantitative analysis.**		
tables.pdf	A summary of all the definitions and files used for the MaxQuant search.	
proteinGroups.txt	File containing all proteins with corresponding label free quantitative information to be imported into the Perseus post-analysis program [15].	
summary.txt parameters.txt	Detailed description of all settings used for the MaxQuant search.	

**Table 1:** Description of file-names in the ProteomeXchange repository PXD002767. MS files were searched using using MaxQuant [10]. Combining fractions of GelMS (1top-4bottom) gel lanes. The number of identified proteins after filtration of typical contaminants and *Phytophthora infestans* is reported.

**File**	**Sample**	**Cultivar**	**Digestion**	**MS system**	**# ID proteins**

Bin_K4_1.raw	Control,	Bintje	GelMS (4 lanes)	Q Exactive	2894
Bin_K4_1b.raw	4 h
Bin_K4_2.raw	(Pooled control leafs (n=3) at 4 h)
Bin_K4_2b.raw
Bin_K4_3.raw
Bin_K4_3b.raw
Bin_K4_4.raw
Bin_K4_4b.raw
Bin_K16_1.raw	Control,	Bintje	GelMS (4 lanes)	Q Exactive	2831
Bin_K16_1b.raw	16 h
Bin_K16_2.raw
Bin_K16_2b.raw
Bin_K16_3.raw
Bin_K16_3b.raw
Bin_K16_4.raw
Bin_K16_4b.raw
Bin_K65_1.raw	Control,	Bintje	GelMS (4 lanes)	Q Exactive	2841
Bin_K65_1b.raw	65 h
Bin_K65_2.raw
Bin_K65_2b.raw
Bin_K65_3.raw
Bin_K65_3b.raw
Bin_K65_4.raw
Bin_K65_4b.raw
Bin_K120_1.raw	Control,	Bintje	GelMS (4 lanes)	Q Exactive	2889
Bin_K120_1b.raw	120 h
Bin_K120_2.raw
Bin_K120_2b.raw
Bin_K120_3.raw
Bin_K120_3b.raw
Bin_K120_4.raw
Bin_K120_4b.raw
BIn_K258_1.raw	Control,	Bintje	GelMS (4 lanes)	Q Exactive	2784
258 h
BIn_K258_1b.raw
BIn_K258_2.raw
BIn_K258_2b.raw
BIn_K258_3.raw
BIn_K258_3b.raw
BIn_K258_4.raw
BIn_K258_4b.raw
Bin_S4_2207_1.raw	*P. infestans* infect,	Bintje	GelMS (4 lanes)	Q Exactive	3057
Bin_S4_2207_2.raw	4 h
Bin_S4_2207_3.raw	(sampling leaf #2207,2208, 2209)
Bin_S4_2207_4.raw
Bin_S4_2208_1.raw
Bin_S4_2208_2.raw
Bin_S4_2208_3.raw
Bin_S4_2208_4.raw
Bin_S4_2209_1.raw
Bin_S4_2209_2.raw
Bin_S4_2209_3.raw
Bin_S4_2209_4.raw
Bin_S16_2258_1.raw	*P. infestans* infect,	Bintje	GelMS (4 lanes)	Q Exactive	3065
Bin_S16_2258_2.raw
16 h
Bin_S16_2258_3.raw
Bin_S16_2258_4.raw
Bin_S16_2259_1.raw
Bin_S16_2259_2.raw
Bin_S16_2259_3.raw
Bin_S16_2259_4.raw
Bin_S16_2260_1.raw
Bin_S16_2260_2.raw
Bin_S16_2260_3.raw
Bin_S16_2260_4.raw
Bin_S65_2309_1.raw	*P. Infestans* infect,	Bintje	GelMS (4 lanes)	Q Exactive	3099
Bin_S65_2309_2.raw
65 h
Bin_S65_2309_3.raw
Bin_S65_2309_4.raw
Bin_S65_2310_1.raw
Bin_S65_2310_2.raw
Bin_S65_2310_3.raw
Bin_S65_2310_4.raw
Bin_S65_2311_1.raw
Bin_S65_2311_2.raw
Bin_S65_2311_3.raw
Bin_S65_2311_4.raw
Bin_S120_2360_1.raw	*P. Infestans* infect,	Bintje	GelMS (4 lanes)	Q Exactive	3149
Bin_S120_2360_2.raw
120 h
Bin_S120_2360_3.raw
Bin_S120_2360_4.raw
Bin_S120_2361_1.raw
Bin_S120_2361_2.raw
Bin_S120_2361_3.raw
Bin_S120_2361_4.raw
Bin_S120_2362_1.raw
Bin_S120_2362_2.raw
Bin_S120_2362_3.raw
Bin_S120_2362_4.raw
Bin_S258_2411_1.raw	*P. Infestans* infect,	Bintje	GelMS (4 lanes)	Q Exactive	3097
Bin_S258_2411_2.raw	258 h
Bin_S258_2411_3.raw
Bin_S258_2411_4.raw
Bin_S258_2412_1.raw
Bin_S258_2412_2.raw
Bin_S258_2412_3.raw
Bin_S258_2412_4.raw
Kur_K4_1.raw	Control,	Kuras	GelMS (4 lanes)	Q Exactive	3006
Kur_K4_1b.raw	4 h
Kur_K4_2.raw
Kur_K4_2b.raw
Kur_K4_3.raw
Kur_K4_3b.raw
Kur_K4_4.raw
Kur_K4_4b.raw
Kur_K16_1.raw	Control,	Kuras	GelMS (4 lanes)	Q Exactive	3004
Kur_K16_1b.raw	16 h
Kur_K16_2.raw
Kur_K16_2b.raw
Kur_K16_3.raw
Kur_K16_3b.raw
Kur_K16_4.raw
Kur_K16_4b.raw
Kur_K65_1.raw	Control,	Kuras	GelMS (4 lanes)	Q Exactive	3177
Kur_K65_1b.raw	65 h
Kur_K65_2.raw
Kur_K65_2b.raw
Kur_K65_3.raw
Kur_K65_3b.raw
Kur_K65_4.raw
Kur_K65_4b.raw
Kur_K120_1.raw	Control,	Kuras	GelMS (4 lanes)	Q Exactive	3144
Kur_K120_1b.raw	120 h
Kur_K120_2.raw
Kur_K120_2b.raw
Kur_K120_3.raw
Kur_K120_3b.raw
Kur_K120_4.raw
Kur_K120_4b.raw
Kur_K258_1.raw	Control,	Kuras	GelMS (4 lanes)	Q Exactive	3122
Kur_K258_1b.raw	258 h
Kur_K258_2.raw
Kur_K258_2b.raw
Kur_K258_3.raw
Kur_K258_3b.raw
Kur_K258_4.raw
Kur_K258_4b.raw
Kur_S4_2231_1.raw	*P. Infestans* infect,	Kuras	GelMS (4 lanes)	Q Exactive	3346
Kur_S4_2231_2.raw
Kur_S4_2231_3.raw	4 h
Kur_S4_2231_4.raw
Kur_S4_2232_1.raw
Kur_S4_2232_2.raw
Kur_S4_2232_3.raw
Kur_S4_2232_4.raw
Kur_S4_2233_1.raw
Kur_S4_2233_2.raw
Kur_S4_2233_3.raw
Kur_S4_2233_4.raw
Kur_S16_2282_1.raw	*P. Infestans* Infect,	Kuras	GelMS (4 lanes)	Q Exactive	3261
Kur_S16_2282_2.raw
16 h
Kur_S16_2282_3.raw
Kur_S16_2282_4.raw
Kur_S16_2283_1.raw
Kur_S16_2283_2.raw
Kur_S16_2283_3.raw
Kur_S16_2283_4.raw
Kur_S16_2284_1.raw
Kur_S16_2284_2.raw
Kur_S16_2284_3.raw
Kur_S16_2284_4.raw
Kur_S65_2333_1.raw	*P. Infestans* infect,	Kuras	GelMS (4 lanes)	Q Exactive	3370
65 h
Kur_S65_2333_2.raw
Kur_S65_2333_3.raw
Kur_S65_2333_4.raw
Kur_S65_2334_1.raw
Kur_S65_2334_2.raw
Kur_S65_2334_3.raw
Kur_S65_2334_4.raw
Kur_S65_2335_1.raw
Kur_S65_2335_2.raw
Kur_S65_2335_3.raw
Kur_S65_2335_4.raw
Kur_S120_2384_1.raw	*P. Infestans* infect,	Kuras	GelMS (4 lanes)	Q Exactive	3406
120 h
Kur_S120_2384_2.raw
Kur_S120_2384_3.raw
Kur_S120_2384_4.raw
Kur_S120_2385_1.raw
Kur_S120_2385_2.raw
Kur_S120_2385_3.raw
Kur_S120_2385_4.raw
Kur_S120_2386_1.raw
Kur_S120_2386_2.raw
Kur_S120_2386_3.raw
Kur_S120_2386_4.raw
Kur_S258_2435_1.raw	*P. Infestans* infect,	Kuras	GelMS (4 lanes)	Q Exactive	3414
Kur_S258_2435_2.raw	258 h
Kur_S258_2435_3.raw
Kur_S258_2435_4.raw
Kur_S258_2436_1.raw
Kur_S258_2436_2.raw
Kur_S258_2436_3.raw
Kur_S258_2436_4.raw
Kur_S258_2437_1.raw
Kur_S258_2437_2.raw
Kur_S258_2437_3.raw
Kur_S258_2437_4.raw
Sar_K4_1.raw	Control,	SarpoMira	GelMS (4 lanes)	Q Exactive	3096
Sar_K4_1b.raw	4 h
Sar_K4_2.raw
Sar_K4_2b.raw
Sar_K4_3.raw
Sar_K4_3b.raw
Sar_K4_4.raw
Sar_K4_4b.raw
Sar_K16_1.raw	Control,	SarpoMira	GelMS (4 lanes)	Q Exactive	2880
Sar_K16_1b.raw	16 h
Sar_K16_2.raw
Sar_K16_2b.raw
Sar_K16_3.raw
Sar_K16_3b.raw
Sar_K16_4.raw
Sar_K16_4b.raw
Sar_K65_1.raw	Control,	SarpoMira	GelMS (4 lanes)	Q Exactive	3099
Sar_K65_1b.raw	65 h
Sar_K65_2.raw
Sar_K65_2b.raw
Sar_K65_3.raw
Sar_K65_3b.raw
Sar_K65_4.raw
Sar_K65_4b.raw
Sar_K120_1.raw	Control,	SarpoMira	GelMS (4 lanes)	Q Exactive	3092
Sar_K120_1b.raw	120 h
Sar_K120_2.raw
Sar_K120_2b.raw
Sar_K120_3.raw
Sar_K120_3b.raw
Sar_K120_4.raw
Sar_K120_4b.raw
Sar_K258_1.raw	Control,	SarpoMira	GelMS (4 lanes)	Q Exactive	3124
Sar_K258_1b.raw	258 h
Sar_K258_2.raw
Sar_K258_2b.raw
Sar_K258_3.raw
Sar_K258_3b.raw
Sar_K258_4.raw
Sar_K258_4b.raw
Sar_S4_2240_1.raw	*P. Infestans* infect,	SarpoMira	GelMS (4 lanes)	Q Exactive	3363
Sar_S4_2240_2.raw
4 h
Sar_S4_2240_3.raw
Sar_S4_2240_4.raw
Sar_S4_2441_1.raw
Sar_S4_2241_2.raw
Sar_S4_2241_3.raw
Sar_S4_2241_4.raw
Sar_S4_2242_2.raw
Sar_S4_2242_3.raw
Sar_S4_2242_4.raw
Sar_S4_2442_1.raw
Sar_S16_2291_1.raw	*P. Infestans* infect,	SarpoMira	GelMS (4 lanes)	Q Exactive	3361
Sar_S16_2291_2.raw
16 h
Sar_S16_2291_3.raw
Sar_S16_2291_4.raw
Sar_S16_2292_1.raw
Sar_S16_2292_2.raw
Sar_S16_2292_3.raw
Sar_S16_2292_4.raw
Sar_S16_2293_1.raw
Sar_S16_2293_2.raw
Sar_S16_2293_3.raw
Sar_S16_2293_4.raw
Sar_S65_2342_1.raw	*P. Infestans* infect,	SarpoMira	GelMS (4 lanes)	Q Exactive	3326
Sar_S65_2342_2.raw	65 h
Sar_S65_2342_3.raw
Sar_S65_2342_4.raw
Sar_S65_2343_1.raw
Sar_S65_2343_2.raw
Sar_S65_2343_3.raw
Sar_S65_2343_4.raw
Sar_S65_2344_1.raw
Sar_S65_2344_2.raw
Sar_S65_2344_3.raw
Sar_S65_2344_4.raw
Sar_S120_2393_1.raw	*P. Infestans* infect,	SarpoMira	GelMS (4 lanes)	Q Exactive	3389
Sar_S120_2393_2.raw
120 h
Sar_S120_2393_3.raw
Sar_S120_2393_4.raw
Sar_S120_2394_1.raw
Sar_S120_2394_2.raw
Sar_S120_2394_3.raw
Sar_S120_2394_4.raw
Sar_S120_2395_1.raw
Sar_S120_2395_2.raw
Sar_S120_2395_3.raw
Sar_S120_2395_4.raw
Sar_S258_2444_1.raw	*P. Infestans* infect,	SarpoMira	GelMS (4 lanes)	Q Exactive	3348
Sar_S258_2444_2.raw
258 h
Sar_S258_2444_3.raw
Sar_S258_2444_4.raw
Sar_S258_2445_1.raw
Sar_S258_2445_2.raw
Sar_S258_2445_3.raw
Sar_S258_2445_4.raw
Sar_S258_2446_1.raw
Sar_S258_2446_2.raw
Sar_S258_2446_3.raw
Sar_S258_2446_4.raw

## References

[1] Kamoun S., Smart C.D. (2005). Late blight of potato and tomato in the genomics era. Plant Dis..

[2] Fry W. (2008). *Phytophthora infestans*: the plant (and R gene) destroyer. Mol. Plant Pathol..

[3] Birch P.R.J. (2009). Towards understanding the virulence functions of RXLR effectors of the oomycete plant pathogen *Phytophthora infestans*. J. Exp. Bot..

[4] Ali A. (2014). Quantitative proteomics and transcriptomics of potato in response to *Phytophthora infestans* in compatible and incompatible interactions. BMC Genom..

[5] Buyel J.F., Twyman R.M., Fischer R. (2015). Extraction and downstream processing of plant-derived recombinant proteins. Biotechnol. Adv..

[6] Lim S. (2012). Protein profiling in potato (*Solanum tuberosum L*.) leaf tissues by differential centrifugation. J. Proteome Res..

[7] Pierpoint W. (1996). The extraction of enzymes from plant tissues rich in phenolic compounds. Methods Mol. Biol..

[8] Lim S. (2013). Proteomics analysis suggests broad functional changes in potato leaves triggered by phosphites and a complex indirect mode of action against *Phytophthora infestans*. J. Proteom..

[9] Stensballe A., Hald S., Bauw G., Blennow A., Welinder K.G. (2008). The amyloplast proteome of potato tuber. FEBS J..

[10] Cox J. (2014). Accurate proteome-wide label-free quantification by delayed normalization and maximal peptide ratio extraction, termed maxLFQ. Mol. Cell. Proteom..

[11] Vizcaíno J.A. (2014). ProteomeXchange provides globally coordinated proteomics data submission and dissemination. Nat. Biotechnol..

[12] Vizcaíno J.A. (2013). The PRoteomics IDEntifications (PRIDE) database and associated tools: status in 2013. Nucleic Acids Res..

[13] Wang R. (2012). PRIDE Inspector: a tool to visualize and validate MS proteomics data. Nat. Biotechnol..

[14] Côté R.G. (2012). The PRoteomics IDEntification (PRIDE) Converter 2 framework: an improved suite of tools to facilitate data submission to the PRIDE database and the ProteomeXchange consortium. Mol. Cell. Proteom..

[15] Cox J. (2014). MaxLFQ allows accurate proteome-wide label-free quantification by delayed normalization and maximal peptide ratio extraction. Mol. Cell. Proteom..

