# Supplementary material for: Time-course investigation of Phytophthora infestans infection of potato leaf from three cultivars by quantitative proteomics
Source: Data Brief. 2015 Dec 17;6:238–48. doi: 10.1016/j.dib.2015.11.069 (PMC4707178; doi:10.1016/j.dib.2015.11.069)
Supplement: Supplementary file 2 — Supplementary material [file mmc2.pdf]

Allan Stensballe

From: pride-support@ebi.ac.uk
Sent: Tuesday, October 13, 2015 12:37 PM
To: Allan Stensballe
Subject: Submission Complete

Dear Allan Stensballe,

We are happy to inform you that your dataset "Time-course Investigation of Phytophthora infestans infection of potato leaf from three cultivars by quantitative proteomics" has been successfully submitted to ProteomeXchange via the PRIDE database. The data is currently private, and can only be accessed with your account or with a single reviewer account that has been created.

Please note that it is essential that you notify us of the first (online) publication of the corresponding manuscript.Otherwise the data will remain inaccessible to readers.

Please add to your manuscript the following sentence (typically in the "Methods" section or just before/in the Acknowledgements):

"The mass spectrometry proteomics data have been deposited to the ProteomeXchange Consortium [1] via the PRIDE partner repository with the dataset identifier PXD002767".

We would recommend you to also include this information in a much abridged form into the abstract itself, e.g. "Data are available via ProteomeXchange with identifier PXD002767."

Submission details:

Project Name: Time-course Investigation of Phytophthora infestans infection of potato leaf from three cultivars by quantitative proteomics
Project accession: PXD002767
Project DOI: Not applicable

Reviewer account details:

Username: reviewer61962@ebi.ac.uk
Password: l2bsVKMu

Don't forget to include these reviewer account details in your manuscript, during the peer review process.

Thank you for choosing PRIDE and ProteomeXchange for the dissemination of your data, and look forward to receiving more of your data in the future.

Regards,

The PRIDE Team
Website: http://www.ebi.ac.uk/pride
Email:pride-support@ebi.ac.uk
Twitter: @pride_ebi

For general ProteomeXchange reference, please use:

[1] Vizcaíno JA, Deutsch EW, Wang R, Csordas A, Reisinger F, Ríos D, Dianes JA, Sun Z, Farrah T, Bandeira N, Binz PA,Xenarios I, Eisenacher M, Mayer G, Gatto L, Campos A, Chalkley RJ, Kraus HJ, Albar JP, Martinez-Bartolomé S, Apweiler R,Omenn GS, Martens L, Jones AR, Hermjakob H (2014). ProteomeXchange provides globally co-ordinated proteomics data submission and dissemination. Nature Biotechnol. 30(3):223-226. PubMed PMID:24727771.

For general PRIDE reference, please use:

[2] Vizcaino JA, Cote RG, Csordas A, Dianes JA, Fabregat A, Foster JM, Griss J, Alpi E, Birim M, Contell J, O'Kelly G,Schoenegger A, Ovelleiro D,

Perez-Riverol Y, Reisinger F, Rios D, Wang R, Hermjakob H (2013). The Proteomics Identifications (PRIDE) database and associated tools: status in 2013. Nucleic Acids Res. 41(D1):D1063-1069. PubMed PMID:23203882.

For PRIDE Inspector reference, please use:

[3] Wang R, Fabregat A, Rios D, Ovelleiro D, Foster JM, Cote RG, Griss J, Csordas A, Perez-Riverol Y, Reisinger F, Hermjakob H, Martens L, Vizcaino JA (2012). PRIDE Inspector: a tool to visualize and validate MS proteomics data.Nat Biotechnol. 30(2):135-7. PubMed PMID: 22318026.

For PRIDE Converter 2 reference please use:

[4] Côté RG, Griss J, Dianes JA, Wang R, Wright JC, van den Toorn HWP, van Breukelen B, Heck AJR, Hulstaert N, Martens L, Reisinger F, Csordas A, Ovelleiro D, Perez-Riverol Y, Barsnes H, Hermjakob H, Vizcaíno JA (2012). The Proteomics IDEntifications (PRIDE) Converter 2 framework: an improved suite of tools to facilitate data submission to the PRIDE database and the ProteomeXchange consortium. Mol Cell Proteomics 11: 1682-1689. PubMed PMID: 22949509.
